# Valvular dysfunction and cardiovascular risk in individuals with opioid use disorder who are maintained on buprenorphine/naloxone: a focus on pan-immune-inflammation value and uric acid-to-albumin ratio

**DOI:** 10.3389/fcvm.2025.1612190

**Published:** 2025-12-18

**Authors:** Hatice Eyiol, Sevdenur Kahraman, Ahmet Taha Sahin, Selver Can, Azmi Eyiol

**Affiliations:** Beyhekim Training and Research Hospital, Konya, Türkiye

**Keywords:** intravenous drug use, buprenorphine/naloxone, pan-immune-inflammation value, uric acid-to-albumin ratio, valvular dysfunction

## Abstract

**Aim:**

Individuals with opioid use disorder (OUD) who are maintained on buprenorphine/naloxone (Suboxone) are at risk of cardiovascular complications, including valvular damage, potentially mediated by chronic immune activation and systemic inflammation. However, the relationship between novel biomarkers such as the Pan-Immune-Inflammation Value (PIV) and uric acid-to-albumin ratio (UAR) and cardiac structural abnormalities in this population remains poorly understood.

**Methods:**

This retrospective cohort study included 140 intravenous Suboxone users and 165 healthy controls, analyzing demographic, clinical, and echocardiographic data collected between January 2023 and January 2025. Laboratory parameters were used to calculate the PIV and the UAR, which were then correlated with echocardiographic findings, including valvular morphology and left ventricular function.

**Results:**

Compared with controls, individuals with OUD + Suboxone had significantly larger right atrial diameters (26 vs. 25 mm, *p* < 0.001) and left atrial diameters (31 vs. 30 mm, *p* = 0.003). Inflammatory markers were markedly elevated, including PIV (232.5 vs. 194.5, *p* = 0.041) and UAR (0.12 vs. 0.11, *p* < 0.001). CRP levels were also higher in the OUD + Suboxone group (6 vs. 3 mg/L, *p* < 0.001). Pulmonary regurgitation was more frequent compared with controls (*p* < 0.001).

**Conclusion:**

This study underscores the potential of PIV and UAR as predictive markers for cardiac pathologies in individuals undergoing buprenorphine/naloxone maintenance therapy, highlighting the need for further validation in larger prospective studies.

## 1 Introduction

Individuals with opioid use disorder (OUD) who are maintained on Suboxone therapy remains a significant global public health issue, associated with high morbidity and mortality rates (1). In 2022, the United States reported a record-high number of opioid-related overdose deaths (81,806), exceeding those of any previous year. Medications used for opioid use disorder (OUD) include buprenorphine, methadone, and extended-release naltrexone. Among these, buprenorphine and methadone are particularly effective, significantly reducing both overdose-related and all-cause mortality; however, they remain markedly underutilized (2, 3).

Cardiovascular complications such as infectious endocarditis, thrombosis, pulmonary hypertension, and structural cardiac damage are commonly observed among individuals who inject drugs. Suboxone—a combination of buprenorphine and naloxone—is widely employed as a maintenance therapy for opioid dependence. Nevertheless, its intravenous misuse has become increasingly prevalent (4). This off-label route of administration compromises the drug's harm-reduction potential and exposes users to additional cardiovascular risks, particularly valvular pathologies (5). Intravenous misuse of buprenorphine/naloxone has been reported in previous studies and has been associated with additional cardiovascular risks (6, 7) In our cohort, all individuals were receiving buprenorphine/naloxone (Suboxone) as prescribed maintenance therapy within a clinical setting; misuse behaviors such as intravenous administration were not documented and were not part of our study design.

Buprenorphine is regarded as a first-line treatment for OUD in both the United States and Canada (8, 9). Its use is associated with a 3.3-fold reduction in overdose mortality and is considered a cornerstone in addressing rising opioid intoxication rates (2, 8). Although the initial formulation was approved in the U.S. in 2002 and in Canada in 2007, the combination product buprenorphine-naloxone remains underused (10, 11). In our study, all patients were using buprenorphine-naloxone (Suboxone). Individuals with opioid use disorder (OUD) who are maintained on Suboxone therapy, valvular heart disease has traditionally been attributed to recurrent endothelial injury, the introduction of foreign particles, and bloodstream infections (12). However, accumulating evidence indicates that beyond microbial insults, chronic immune activation, oxidative stress, and systemic inflammation play critical roles in the pathogenesis of valvular damage (13). These mechanisms may be particularly prominent in IV Suboxone users due to the pharmacological properties of the compound and the repeated vascular trauma associated with injection practices (14). Nevertheless, the extent to which systemic immune-inflammatory responses are associated with echocardiographic findings in this population remains unclear.

In this context, emerging composite biomarkers offer promising tools for evaluating systemic inflammation and oxidative status. The Pan-Immune-Inflammation Value (PIV), a recently proposed index derived from neutrophil, monocyte, platelet, and lymphocyte counts, provides a comprehensive reflection of a patient's pro-inflammatory and immune status (15, 16). Unlike isolated inflammatory markers, PIV has been associated with disease severity and prognosis in various cardiovascular and oncologic conditions. Similarly, the uric acid-to-albumin ratio (UAR) serves as a dual marker that captures both oxidative stress and systemic inflammation, as elevated uric acid and reduced serum albumin levels have been independently linked to adverse cardiovascular outcomes (17).

Despite their clinical relevance, studies investigating the relationship between these novel biomarkers and cardiac structural abnormalities in individuals with opioid use disorder (OUD) who are maintained on Suboxone therapy are scarce. To date, no study has specifically focused on IV Suboxone users, a growing and underexplored subgroup of substance users. Understanding how biomarkers such as PIV and UAR correlate with valvular involvement, left ventricular function, and tissue Doppler parameters could enhance early risk stratification and offer insights into non-infectious mechanisms contributing to cardiac pathology in this population. Moreover, such biomarkers may aid in distinguishing whether valvular dysfunction in these patients arises from mechanical vs. infectious etiologies within an inflammatory milieu.

This study aims to investigate the relationship between PIV and UAR levels and echocardiographic valvular abnormalities in patients with a history of patients receiving prescribed Suboxone maintenance therapy. By integrating laboratory and imaging data, we seek to clarify whether systemic immune-inflammatory and oxidative profiles predict structural valvular alterations. Our findings may support the use of biomarker-guided cardiovascular assessment approaches in individuals with opioid use disorder (OUD) and contribute to a better understanding of OUD-related cardiac complications.

## 2 Material and methods

### 2.1 Compliance with ethical standards

This study was conducted in accordance with the ethical standards of the Declaration of Helsinki and approved by the Institutional Ethics Committee of Necmettin Erbakan University, Konya, Türkiye. The ethics approval was granted during the committee meeting held on May 3, 2024, with decision number 2024/4958. No artificial intelligence-assisted technologies were used at any stage of the study.

### 2.2 Study design

This study was designed as a retrospective cohort analysis including 140 patients with a history of prescribed Suboxone maintenance therapy and 165 healthy controls between January 2023 and January 2025. Patient data were obtained from electronic hospital medical records and included demographic characteristics, clinical histories, laboratory findings, and transthoracic echocardiographic results. Laboratory parameters were used to calculate the Pan-Immune-Inflammation Value (PIV) and the uric acid-to-albumin ratio (UAR), while echocardiographic data were used to assess valvular morphology, left ventricular function, and tissue Doppler parameters. The duration of prescribed Suboxone maintenance therapy was recorded for all patients; however, the duration of opioid use disorder (OUD) prior to Suboxone initiation could not be reliably retrieved from electronic hospital records. All individuals in the OUD + Suboxone group were receiving prescribed buprenorphine/naloxone (Suboxone) as maintenance therapy within a supervised clinical setting; misuse behaviors such as intravenous administration were not documented and were not part of our study design.

### 2.3 Patient evaluation and follow-up

Patients included in the study were individuals aged between 18 and 65 years with documented intravenous Suboxone use for opioid maintenance therapy for at least 3 months. Patients were eligible if complete echocardiographic data and laboratory parameters were available during the same clinical evaluation period.

Exclusion criteria included known structural heart disease prior to Suboxone initiation, significant coronary artery disease, left ventricular systolic dysfunction, uncontrolled hypertension or diabetes mellitus, chronic hepatic or renal failure, malignancies, systemic inflammatory or autoimmune diseases, and active infections. Cases with suboptimal echocardiographic imaging or missing laboratory data were also excluded.

Detailed clinical histories, including opioid use duration and route, comorbidities, and medication profiles, were reviewed for all participants. All patients underwent standard transthoracic echocardiographic evaluation using standard imaging protocols to assess valvular thickness, calcification, regurgitation, stenosis, and left ventricular and atrial dimensions and functions.

Blood samples were analyzed for complete blood count (including neutrophil, lymphocyte, monocyte, and platelet counts) and biochemical parameters such as serum uric acid and albumin levels. PIV was calculated using the formula: (neutrophil × monocyte × platelet)/lymphocyte. UAR was calculated by dividing the serum uric acid level by the serum albumin level. These markers were analyzed in relation to echocardiographic findings.

### 2.4 Echocardiographic assessment

Pulsed-wave spectral Doppler echocardiography was performed at an end-expiratory phase using a 5-mm sample volume placed at the tips of the mitral leaflets, aligned with the inflow, at a sweep speed of 100 mm/s. Tissue Doppler imaging (TDI) was performed at the level of the mitral annulus using low wall filter settings and minimal gain to optimize the signal; Nyquist limits were set to 15–20 cm/s with a frame rate of 200 Hz.

Each spectral tracing was downloaded for offline analysis using the HDILab software program by a single investigator experienced in tissue Doppler evaluation and blinded to all clinical and echocardiographic data. Peak systolic (s′) and early diastolic (e′) velocities were measured from both the septal and lateral mitral annulus. The average of three consecutive cardiac cycles was used for each measurement. The ratio of transmitral E-wave velocity to averaged e′ (E/e′) was calculated. Intra-observer variability of echocardiographic measurements was assessed at the beginning of the study and at regular intervals throughout the study period.

### 2.5 Statistical analysis

All statistical analyses were performed using SPSS software version 27.0 (IBM Corp., Chicago, IL, USA). The normality of distribution for continuous variables was assessed using the Kolmogorov–Smirnov test, histogram analysis, skewness/kurtosis values, and Q–Q plots. Categorical variables were expressed as frequencies (*n*) and percentages (%), while continuous variables were presented as mean ± standard deviation (SD) or median and interquartile range [IQR; median (minimum–maximum)], depending on the distribution. Group comparisons for normally distributed variables were conducted using the independent samples *t*-test, while the Mann–Whitney *U* test was used for non-normally distributed variables. Homogeneity of variances was evaluated with Levene's test. For comparisons involving more than two groups, the Kruskal–Wallis *H* test was used, and pairwise *post hoc* analyses were conducted using the Dunn test.

Associations between categorical variables were assessed using the Pearson chi-square test or Fisher's exact test, where appropriate. The potential impact of variables on binary outcomes was examined through univariate and multivariate logistic regression analyses. Receiver operating characteristic (ROC) curve analyses were performed to determine the optimal cutoff values for selected parameters that best discriminated between the study groups. Correlations between continuous variables were evaluated using Pearson or Spearman correlation coefficients, depending on data distribution. All statistical tests were two-tailed, and a *p*-value <0.05 was considered statistically significant.

## 3 Results

### 3.1 Demographic and baseline characteristics

Table 1 presents a comparison of patient characteristics, vital signs, ECG, and echocardiographic parameters between the control group and individuals with opioid use disorder (OUD) who are maintained on Suboxone therapy. The mean age was 28.8 ± 5.5 years in the control group and 28.7 ± 5.5 years individuals with OUD + Suboxone group. Right atrial and right ventricular diameters were significantly larger in individuals with OUD + Suboxone compared to the controls (OUD + Suboxone vs. controls, *p* < 0.001). Septal A’, lateral A’, and septal E/e′ ratios were also significantly higher in individuals with OUD + Suboxone (OUD + Suboxone vs. controls, *p* = 0.006, *p* = 0.027, and *p* = 0.049, respectively).

**Table 1:** Comparison of patient characteristics, vital signs, ECG and ECHO parameters between control and individuals with opioid use disorder (OUD) who are maintained on Suboxone therapy.

Findings	Sample		Overall (general)^a^	*p*
	Control group (165, %54.1)	OUD (140, %45.9)		
	Distribution^a^			
Age	28.8 ± 5.5	28.7 ± 5.5	28.8 ± 5.5	0.849*
Gender				
Male	162 (%98.18)	138 (%98.57)	300 (%98.36)	>0.999**
Female	3 (%1.82)	2 (%1.43)	5 (%1.64)	
Height	174.6 ± 7.6	174.7 ± 7.5	174.6 ± 7.5	0.903*
BSA	1.79 (1.34–2.63)	1.79 (1.34–2.63)	1.79 (1.34–2.63)	0.943***
Weight	65 (42–130)	65 (42–130)	65 (42–130)	0.915***
Pulse pressure	41 (37–50)	41 (37–51)	41 (37–51)	0.473***
Systolic BP	110.1 ± 5.9	110.5 ± 5.9	110.3 ± 5.9	0.555*
Diastolic BP	69.4 ± 5.7	69.5 ± 5.7	69.4 ± 5.7	0.85*
Mean BP	83 ± 5.7	83.2 ± 5.7	83.1 ± 5.7	0.741*
Left ventricle end diastolic diameter	44.6 ± 2.5	44.7 ± 2.5	44.6 ± 2.5	0.732*
Left ventricle end systolic diameter	27.8 ± 1.5	27.9 ± 1.5	27.9 ± 1.5	0.793*
Aortic diameter	24.9 ± 2.3	24.8 ± 2.2	24.8 ± 2.2	0.883*
Left atrium diameter	28 (26–36)	28 (26–36)	28 (26–36)	0.943***
Pulmonary artery compressor	5 (5–38)	23 (5–38)	16 (5–38)	0.915***
Intraventricular septum wall thickness	10 (9–11)	10 (9–11)	10 (9–11)	0.473***
Posterior wall thickness	9 (8–10)	9 (8–10)	9 (8–10)	0.902***
Right atrium diameter	25 (21–31)	26 (22–35)	25 (21–35)	**<0** **.** **001**
Right ventricle diameter	24 ± 2.0	25.8 ± 2.6	24.8 ± 2.5	**<0** **.** **001** *
TAPSE	24.1 ± 1.1	24.2 ± 1.1	24.2 ± 1.1	0.731*
Vena cava inferior diameter (expirium diameter)	1.53 ± 0.09	1.58 ± 0.1	1.56 ± 0.1	**<0** **.** **001** *
E velocity	82 ± 9	82 ± 11	82 ± 10	0.796*
A velocity	59 (30–101)	59 (30–101)	59 (30–101)	0.507***
Septal e’ velocity	113 (67–139)	112.5 (67–139)	113 (67–139)	0.145***
Septal a’ velocity	83 (57–122)	86 (57–155)	85 (57–155)	**0** **.** **006** ***
Septal E/e’ ratio	0.71 (0.43–1.31)	0.75 (0.43–1.31)	0.73 (0.43–1.31)	**0** **.** **049** ***
Lateral e’ velocity	155 (103–186)	155 (78–186)	155 (78–186)	0.513***
Lateral a’ velocity	118 (74–162)	122 (74–198)	119 (74–198)	**0** **.** **027** ***
Lateral E/e’ ratio	0.53 (0.35–0.85)	0.53 (0.34–0.97)	0.53 (0.34–0.97)	0.326***
Infrarenal abdominal aortic diameter (mm)	19.7 ± 0.4	19.7 ± 0.4	19.7 ± 0.4	0.925*
s’ (mm/sn)	67.2 ± 2.3	67.1 ± 2.3	67.1 ± 2.3	0.661*

All patients with EF values of 60% were excluded from the analysis. Data are presented as mean ± SD or median (IQR). Between-group comparisons were performed using independent *t*-test or Mann–Whitney *U* test as appropriate. Categorical variables were compared using chi-square test. TAPSE, tricuspid annular plane systolic excursion; ECG, electrocardiogram; ECHO, echocardiography; IV, intravenous; OUD group, Individuals diagnosed with opioid use disorder (OUD) receiving prescribed suboxone maintenance therapy.

Bold values indicate statistical significance (*p* < 0.05).

a Parameters are expressed as IQR (Interquartile Range) [median, min and max].

* Independent *t*-test.

** Fisher's exact test.

*** Mann–Whitney *U* test.

### 3.2 Laboratory parameters

Table 2 shows the comparison of laboratory parameters and specific ratio-based values between the two groups. White blood cell and neutrophil counts were significantly elevated in individuals with OUD + Suboxone (OUD + Suboxone vs. controls, *p* < 0.001). Likewise, levels of troponin, C-reactive protein (CRP), and uric acid were significantly higher in individuals with OUD + Suboxone (OUD + Suboxone vs. controls, all *p* < 0.001). Fibrinogen and glucose levels were also significantly elevated in this group (OUD + Suboxone vs. controls, *p* = 0.033 and *p* = 0.003, respectively). Both PIV (platelet-to-lymphocyte ratio) and UAR (uric acid to albumin ratio) values were significantly higher in individuals with OUD + Suboxone (OUD + Suboxone vs. controls, *p* = 0.041 and *p* < 0.001, respectively).

**Table 2:** Comparison of laboratory parameters and proportional values between control and individuals with opioid use disorder (OUD) who are maintained on Suboxone therapy.

Findings	Sample		Overall (general) ^a^	*p*
	Control Group (165, %54.1)	OUD (140, %45.9)		
	Distribution^a^			
WBC	6.78 ± 1.1	7.63 ± 1.77	7.17 ± 1.51	**0** **.** **001** *
Platelet	253.5 ± 68.7	248.7 ± 68.4	251.3 ± 68.5	0.548*
Lymphocyte	2.4 ± 0.58	2.42 ± 0.76	2.41 ± 0.67	0.829*
Monocyte	0.54 ± 0.18	0.54 ± 0.19	0.54 ± 0.19	0.934*
Neutrophil	3.73 ± 0.73	4.52 ± 1.47	4.09 ± 1.2	**<0** **.** **001** *
Hemoglobin	14.6 ± 1.2	14.4 ± 1.4	14.5 ± 1.3	0.141*
RDW	13.8 (11.9–19.9)	13.9 (11.9–19.9)	13.8 (11.9–19.9)	0.796**
Albumin	43.7 (40.2–52.2)	43.6 (4.4–52.2)	43.6 (4.4–52.2)	0.125**
Uric Acid	4.8 (2.5–6.6)	5.2 (2.5–9.4)	5 (2.5–9.4)	<0.001**
Triglyceride	109 (26–248)	110.5 (26–801)	110 (26–801)	0.163**
Ferritin	42 (6–201)	43.5 (6–434)	43 (6–434)	0.479**
D-dimer	240 (190–570)	234.5 (190–2,070)	235 (190–2,070)	0.370**
CRP	3 (1–10)	6 (0.87–98)	4 (0.87–98)	**<0** **.** **001** **
Troponin	2.5 (2.5–2.53)^b^	2.5 (2.5–12)^b^	2.5 (2.5–12)	**<0** **.** **001** **
LDL	86.2 ± 28.4	86.1 ± 30.5	86.1 ± 29.3	0.965*
HDL	43.9 ± 10.1	43.5 ± 10	43.7 ± 10	0.703*
Fibrinogen	2.6 ± 0.46	2.73 ± 0.59	2.66 ± 0.52	**0** **.** **033** *
Glucose	87 ± 9.8	91.5 ± 15.3	89.1 ± 12.8	**0** **.** **003** *
TSH	2.18 (0.63–4.6)	2.07 (0.01–29.2)	2.15 (0.01–29.2)	0.151**
PIV	194.54 (14.68–677.65)	232.5 (14.7–1,162.7)	211.8 (14.7–1,162.7)	**0** **.** **041** **
UAA	0.11 (0.05–0.16)	0.12 (0.05–1.14)	0.113 (0.054–1.136)	**<0** **.** **001** **

Data are presented as mean ± SD or median (IQR). Between-group comparisons were performed using independent *t*-test or Mann–Whitney *U* test as appropriate. Categorical variables were compared using chi-square test. WBC, white blood cell; RDW, red blood cell distribution width; CRP, C-reactive protein; TSH, thyroid Stimulating Hormone; HDL, high density lipoprotein; LDL, low density lipoprotein; PIV, pan-immune inflammatory value; UAR, uric acid/albumin ratio; IV, intravenous; OUD group, individuals diagnosed with opioid use disorder (OUD) receiving prescribed suboxone maintenance therapy.

Bold values indicate statistical significance (*p* < 0.05).

a Parameters are expressed as IQR (Interquartile Range) [median, min and max].

b Mean rank (troponin): control group = 142.36; iv drug dependency = 165.54.

* Independent *t-*test.

** Mann–Whitney *U* test.

### 3.3 Echocardiographic structural measurements

Table 3 summarizes the comparison of valvular morphology and severity of regurgitations between the two groups. Myxomatous and rheumatic mitral valve structures were more frequently observed in individuals with OUD + Suboxone (11% and 42%, respectively), whereas the control group predominantly had normal valvular anatomy (100%). The significant differences between the groups were particularly observed in the prevalence of normal and rheumatic valve morphologies. Regarding the aortic valve, a significant difference was also noted in the proportion of normal and rheumatic valve structures, with rheumatic valves being more common among individuals with OUD + Suboxone (OUD + Suboxone vs. controls, 2.68%; *p* = 0.02). In terms of valvular insufficiencies, the frequencies and severities of mitral, aortic, tricuspid, and pulmonary regurgitations were significantly higher in the individuals with OUD + Suboxone (OUD + Suboxone vs. controls, *p* < 0.001, *p* = 0.02, *p* = 0.002, and *p* < 0.001, respectively). We did not evaluate misuse behaviors, as all individuals were receiving prescribed Suboxone as maintenance therapy.

**Table 3:** Comparison of valve structure and insufficiency degrees between control and individuals with opioid use disorder (OUD) who are maintained on Suboxone therapy.

Findings		Sample		Overall (general)^a^	*p*
		Control Group (165, %54.1)	OUD (140, %45.9)		
		Distribution^a^			
Mitral valve	Myxomatous	0 (%0)	11 (%7.86)	11 (%3.61)	**<0** **.** **001** *
	Normal^b^	165 (%100)	87 (%62.14)	252 (%82.62)	
	Rheumatic^b^	0 (%0)	42 (%30)	42 (%13.77)	
Aortic valve structure	Myxomatous	0 (%0)	1 (%0.71)	1 (%0.33)	**0** **.** **02** **
	Normal	165 (%100)	135 (%96.43)	300 (%98.36)	
	Rheumatic	0 (%0)	4 (%2.86)	4 (%1.31)	
Tricuspid valve structure	Myxomatous	0 (%0)	3 (%2.14)	3 (%0.98)	**0.096** **
	Normal	165 (%100)	137 (%97.86)	302 (%99.02)	
Mitral regurgitation	1^b^	0 (%0)	8 (%5.71)	8 (%2.62)	**<0** **.** **001** **
	Minimal^b^	65 (%39.39)	77 (%55)	142 (%46.56)	
	Mild	34 (%20.61)	41 (%29.29)	75 (%24.59)	
	None^b^	66 (%40)	14 (%10)	80 (%26.23)	
Aortic regurgitation	1	0 (%0)	2 (%1.43)	2 (%0.66)	**0** **.** **02** **
	Minimal	0 (%0)	1 (%0.71)	1 (%0.33)	
	Mild	0 (%0)	2 (%1.43)	2 (%0.66)	
	None^b^	165 (%100)	135 (%96.43)	300 (%98.36)	
Tricuspid regurgitation	1^b^	0 (%0)	9 (%6.43)	9 (%2.95)	**0** **.** **002** **
	Minimal	70 (%42.42)	60 (%42.86)	130 (%42.62)	
	Mild	95 (%57.58)	71 (%50.71)	166 (%54.43)	
Pulmonary regurgitation	1	0 (%0)	18 (%12.86)	18 (%5.9)	**<0** **.** **001** *
	Minimal	18 (%10.91)	13 (%9.29)	31 (%10.16)	
	Mild	147 (%89.09)	109 (%77.86)	256 (%83.93)	

Data are presented as mean ± SD or median (IQR). Between-group comparisons were performed using independent *t*-test or Mann–Whitney *U* test as appropriate. Categorical variables were compared using chi-square test. IV, intravenous; OUD group, Individuals diagnosed with opioid use disorder (OUD) receiving prescribed suboxone maintenance therapy.

Bold values indicate statistical significance (*p* < 0.05).

a Parameters are expressed as frequency (N) and percentage (%).

b Rows (subcategories) with significant proportional differences in the confusion matrix are marked.

* Pearson chi-square analysis.

** Fisher's exact test.

### 3.4 Valvular morphology

Table 4 displays the correlation between age, clinical characteristics, vital parameters, and echocardiographic findings. A strong positive correlation was observed between age and infrarenal aortic diameter, as well as between age and inferior vena cava (IVC) diameter.

**Table 4:** Correlation relationships of age, patient characteristics, vital parameters and ECHO findings.

Parameters	Age^a^		Height^a^		Weigh		BSA		Dependency period (months)	
	rho/r	*p*	rho/r	*p*	rho/r	*p*	rho/r	*p*	rho/r	*p*
LVEDD^a^	0.134	0.115	0.159	0.06	0.197	**0** **.** **02**	0.19	**0** **.** **025**	−0.05	0.556
LVESD^a^	0.075	0.376	0.137	0.107	0.152	0.074	0.129	0.128	−0.096	0.262
Aortic diameter^a^	0.145	0.087	0.228	**0** **.** **007**	0.257	**0** **.** **002**	0.265	**0** **.** **002**	−0.048	0.572
Left atrium diameter	0.247	**0** **.** **003**	0.134	0.114	0.372	**<0** **.** **001**	0.357	**<0** **.** **001**	−0.091	0.284
PAP	0.056	0.513	0.024	0.782	0.043	0.611	0.063	0.46	0.106	0.212
IVSWT	0.324	**<0** **.** **001**	0.075	0.381	0.308	**<0** **.** **001**	0.264	**0** **.** **002**	0.078	0.362
PDT	0.324	**<0** **.** **001**	0.075	0.381	0.308	**<0** **.** **001**	0.264	**0** **.** **002**	0.078	0.362
Right atrium diameter	0.226	**0** **.** **007**	0.132	0.12	0.306	**<0** **.** **001**	0.287	**0** **.** **001**	0.136	0.108
Right ventricular diameter^a^	0.176	**0** **.** **037**	0.19	**0** **.** **024**	0.308	**<0** **.** **001**	0.294	**<0** **.** **001**	0.161	0.057
TAPSE^a^	−0.124	0.144	−0.073	0.392	−0.084	0.324	−0.09	0.293	0.065	0.447
VCI expirium diameter^a^	0.753	**<0** **.** **001**	0.172	**0** **.** **042**	0.138	0.105	0.138	0.105	0.018	0.833
E velocity^a^	−0.199	**0** **.** **019**	0.18	**0** **.** **033**	0.035	0.682	0.071	0.402	−0.114	0.178
A velocity	−0.094	0.272	0.137	0.107	0.09	0.292	0.131	0.124	−0.034	0.689
Septal e’ velocity	−0.232	**0** **.** **006**	0.005	0.952	−0.133	0.118	−0.098	0.247	0.036	0.671
Septal a’ velocity	0.321	**<0** **.** **001**	−0.056	0.508	0.137	0.107	0.092	0.278	0.008	0.93
Septal E/E’ ratio	0.029	0.737	0.079	0.352	0.152	0.072	0.157	0.063	−0.069	0.42
Lateral e’ velocity	−0.139	0.101	−0.033	0.703	−0.037	0.667	−0.028	0.739	0.032	0.71
Lateral a’ velocity	0.206	**0** **.** **014**	−0.072	0.4	0.138	0.103	0.096	0.257	−0.092	0.28
Lateral E/e’ Ratio	−0.048	0.573	0.072	0.4	0.089	0.297	0.104	0.223	−0.112	0.189
IAA diameter^a^	0.935	**<0** **.** **001**	−0.064	0.455	0.115	0.177	0.089	0.294	0.105	0.218
s’ (mm/sn)^a^	−0.022	0.799	−0.221	**0** **.** **009**	−0.075	0.376	−0.092	0.28	−0.125	0.142

Spearman correlation analysis (correlation coefficient: rho) was used for the correlation relationships of the parameters marked with (^a^), and Pearson correlation analysis (correlation coefficient: *r*) was used for the correlation relationships of the other parameters with each other. Data are presented as mean ± SD or median (IQR). Between-group comparisons were performed using independent *t*-test or Mann–Whitney *U* test as appropriate. Categorical variables were compared using chi-square test. IVSWT, interventricular septum wall thickness; LVEDD, left ventricular end-diastolic diameter; LVESD, left ventricular end-systolic diameter; TAPSE, tricuspid annular plane systolic excursion; VCI, vena cava inferior, IAA, infrarenal abdominal aorta; PDK, posterior wall thickness; PAP, pulmonary artery pressure.

Bold values indicate statistical significance (*p* < 0.05).

### 3.5 Univariate logistic regression outcomes

Table 5 shows the results of univariate logistic regression (LR) analysis investigating the association of age, sex, echocardiographic parameters, and laboratory values with OUD + Suboxone. Increased pulmonary artery pressure, septal E/e’ ratio, right atrial and right ventricular diameters, IVC diameter, PIV, UAR, triglyceride, D-dimer, CRP, fibrinogen, and glucose levels were all positively associated with individuals with OUD + Suboxone.

**Table 5:** Evaluation of the relationships between age, gender, echocardiography findings and laboratory parameters and individuals with opioid use disorder (OUD) who are maintained on Suboxone therapy using univariate logistic regression (LR) analysis.

Parameters	*β*	Nagelkerke *R*^2^	*p*	OR	95% CI	
					Lower limit	Upper limit
Age	−0.004	<0.001	0.848	0.996	0.956	1.038
Gender	−0.245	<0.001	0.790	0.783	0.129	4.751
Pulmonary artery pressure	0.185	0.4940	**<0** **.** **001**	1.203	1.157	1.250
Septal E/e’ ratio	2.383	0.0233	**0** **.** **024**	10.835	1.368	85.795
Lateral E/e'	2.967	0.0188	**0** **.** **042**	19.443	1.110	340.627
s’ (mm/sn)	−0.022	<0.001	0.659	0.978	0.887	1.079
Right atrium diameter	0.324	0.1723	**<0** **.** **001**	1.382	1.243	1.538
Right ventricular diameter	0.329	0.1697	**<0** **.** **001**	1.390	1.246	1.549
TAPSE	0.035	<0.001	0.730	1.035	0.850	1.262
VCI diameter	5.324	0.0815	**<0** **.** **001**	205.111	16.458	2,556.285
IAA diameter	−0.031	<0.001	0.924	0.970	0.515	1.825
PIV	0.002	0.0452	**0** **.** **002**	1.002	1.001	1.004
UAR^a^	3.091	0.101	**<0** **.** **001**	21.998	5.701	84.880
Triglyceride	0.006	0.0398	**0** **.** **007**	1.006	1.002	1.010
Ferritin	0.005	0.0162	0.075	1.005	1.000	1.011
D-dimer	0.002	0.0707	**0** **.** **002**	1.002	1.001	1.004
CRP	0.390	0.3450	**<0** **.** **001**	1.478	1.307	1.671
LDL	0.000	<0.001	0.965	1.000	0.992	1.008
HDL	−0.004	<0.001	0.702	0.996	0.973	1.018
Fibrinogen	0.487	0.0207	**0** **.** **031**	1.627	1.044	2.534
Glucose	0.034	0.0464	**0** **.** **003**	1.035	1.012	1.059
TSH	0.076	0.0066	0.265	1.079	0.944	1.234

OR, odd ratio; CI, Confidence interval. Data are presented as mean ± SD or median (IQR). Between-group comparisons were performed using independent *t*-test or Mann–Whitney *U* test as appropriate. Categorical variables were compared using chi-square test. CRP, C-reactive protein; TSH, thyroid stimulating hormone; HDL, high-density lipoprotein; LDL, low-density lipoprotein; PIV, pan-immune inflammatory value; UAR, uric acid/albumin ratio; IV, intravenous; TAPSE, tricuspid annular plane systolic excursion; VCI, vena cava inferior; İAA, infrarenal abdominal aorta.

Bold values indicate statistical significance (*p* < 0.05).

a The relevant parameters showing excessively skewed distribution were subjected to logarithmic transformation and made suitable for the model.

### 3.6 Multivariate logistic regression outcomes

Table 6 presents the multivariate logistic regression (LR) analysis evaluating multiple models to determine independent predictors of OUD + Suboxone. In the comprehensive model, septal E/e’, right atrial diameter, UAR, and D-dimer levels remained statistically significant, indicating their independent association OUD + Suboxone.

**Table 6:** Evaluation of the relationships between parameters and individuals with opioid use disorder (OUD) who are maintained on Suboxone therapy using multivariate logistic regression (LR) analysis.

Nagelkerke *R*^2^ = 0.492					
Parameters	*β*	*p*	OR	95% CI	
				Lower limit	Upper limit
Septal E/e’ ratio	4.958	**<0.001**	142.4	10.27	1,972.5
Right atrium diameter	0.497	**<0.001**	1.64	1.417	1.906
PIV	0.002	0.062	1.00	1.000	1.004
UAR^a^	2.265	**0.008**	9.64	1.804	51.45
D-dimer	0.003	**0.019**	1.003	1.000	1.005
Fibrinogen	−0.391	0.237	0.677	0.354	1.292

Parameters that do not meet the Box-tidwell assumption (pulmonary artery pressure, triglyceride, CRP and glucose) were not included in the multiple model. Due to the multicollinearity problem between some parameters, the Lateral E/E’ ratio, right atrium and VCI diameters, which are incompatible with the model, were excluded from the analyses. Data are presented as mean ± SD or median (IQR). Between-group comparisons were performed using independent *t*-test or Mann–Whitney *U* test as appropriate. Categorical variables were compared using chi-square test. OR, odd ratio; CI, confidence interval; PIV, pan-immune inflammatory value; UAR, uric acid/albumin ratio; IV, intravenous.

Bold values indicate statistical significance (*p* < 0.05).

a The relevant parameters showing excessively skewed distribution were subjected to logarithmic transformation and made suitable for the model.

### 3.7 ROC analysis

Table 7 provides the results of ROC analysis and cut-off values for parameters associated with OUD + Suboxone. Pulmonary artery pressure had the highest diagnostic performance at a cut-off value of ≥15.5 mmHg, with an AUC of 0.864 (95% CI: 0.820–0.907), sensitivity of 87.1%, and specificity of 78.8% (OUD + Suboxone vs. controls, *p* < 0.001). Right atrial and ventricular diameters, IVC diameter, PIV, UAR, CRP, and glucose levels were also found to be statistically significant predictors.

**Table 7:** ROC analysis of parameters associated with iv drug dependence and definition of cut-off values.

Parameters	AUC (%95 CI)	Cut-off	*p*	Sensitivity (%)	Specificity (%)
Pulmonary artery pressure	0.864 (0.820–0.907)	≥15.5	**<0.001**	%87.1	%78.8
Septal E/e’ ratio	0.565 (0.50–0.631)	≥0.732	**0.049**	%56.4	%55.8
Lateral E/e’	0.533 (0.467–0.598)	≥0.526	0.326	%50.7	%50.3
Right atrium diameter	0.696 (0.637–0.756)	≥25.5	**<0.001**	%60.7	%67.9
Right ventricular diameter	0.697 (0.637–0.756)	≥24.5	**<0.001**	%61.4	%67.9
VCI diameter	0.644 (0.582–0.707)	≥1.565	**<0.001**	%57.9	%60.6
PIV	0.568 (0.502–0.634)	≥211.8	**0.041**	%54.3	%53.7
UAR	0.669 (0.607–0.732)	≥0.113	**<0.001**	%60.0	%60.6
Triglyceride	0.546 (0.480–0.613)	≥109.5	0.163	%50.7	%50.3
D-dimer	0.529 (0.463–0.595)	≥237.5	0.380	%49.3	%49.7
CRP	0.719 (0.655–0.784)	≥5.4	**<0.001**	%56.4	%98.2
Fibrinogen	0.553 (0.488–0.618)	≥2.67	0.111	%52.9	%53.9
Glucose	0.600 (0.534–0.665)	≥89.5	**0.003**	%57.1	%53.3

Data are presented as mean ± SD or median (IQR). Between-group comparisons were performed using independent *t*-test or Mann–Whitney *U* test as appropriate. Categorical variables were compared using chi-square test. AUC, area under curve; ROC, receiver operating characteristic; CI, confidence interval reference; category, control group; PIV, pan-immune inflammatory value; UAR, uric acid/albumin ratio; VCI, vena cava inferior; CRP, C-reactive protein.

Bold values indicate statistical significance (*p* < 0.05).

### 3.8 Correlation and regression analyses (PIV & UAR)

Table 8 compares UAR and PIV values across different valvular pathologies. Among patients with myxomatous and rheumatic mitral valves, PIV values were significantly higher compared to those with normal valves (OUD + Suboxone vs. controls, *p* = 0.013). Additionally, PIV values were significantly elevated in patients with mild mitral regurgitation compared to those with trace or no mitral regurgitation (OUD + Suboxone vs. controls, *p* = 0.02). As the degree of pulmonary regurgitation increased, both UAR and PIV values significantly increased (OUD + Suboxone vs. controls, *p* < 0.001 and *p* = 0.01, respectively). Pairwise comparisons between groups are summarized in Figure 3.

**Table 8:** Comparison of UAR and PIV values according to valve pathologies.

Findings		Frequency (*N*)	UAR (Median, min–max)	*p* *	PIV (Median, min–max)	*p* *
Mitral valve	Myxomatous	11 (%3.61)	0.115 (0.066–0.205)	0.316	214.22 (121.47–932.73)	**0** **.** **013**
	Normal	252 (%82.62)	0.113 (0.054–1.136)		200.61 (14.68–732.46)	
	Rheumatic	42 (%13.77)	0.119 (0.054–0.158)		281.61 (66.85–1,162.73)	
Mitral regurgitation	1	8 (%2.62)	0.12 (0.054–0.157)	0.115	195.73 (105.48–745.86)	**0** **.** **002**
	Minimal	142 (%46.56)	0.113 (0.066–1.136)		184.15 (14.68–75.9)	
	Mild	75 (%24.59)	0.108 (0.066–0.205)		263.3 (68.65–1,162.73)	
	None	80 (%26.23)	0.113 (0.054–0.191)		198.46 (14.68–704.09)	
Tricuspid regurgitation	1	9 (%2.95)	0.119 (0.102–0.135)	0.431	510.82 (66.85–745.86)	0.084
	Minimal	130 (%42.62)	0.112 (0.066–1.136)		237.11 (42.51–932.73)	
	Mild	166 (%54.43)	0.114 (0.054–0.228)		192.91 (14.68–1,162.73)	
Pulmonary regurgitation	1	18 (%5.9)	0.118 (0.054–0.138)	**<0** **.** **001**	398.45 (98.6–745.86)	**0** **.** **01**
	Minimal	31 (%10.16)	0.101 (0.066–0.124)		139.71 (102.64–649.13)	
	Mild	256 (%83.93)	0.115 (0.054–1.136)		212.39 (14.68–1,162.73)	

Data are expressed as interquartile range (IQR) [median, minimum and maximum]. Data are presented as mean ± SD or median (IQR). Between-group comparisons were performed using independent *t*-test or Mann–Whitney *U* test as appropriate. Categorical variables were compared using chi-square test. PIV, pan-immune inflammatory volue; UAR, uric acid/albumin ratio; IV, intravenous.

Bold values indicate statistical significance (*p* < 0.05).

* Kruskal–Wallis *H* test.

**Figure 1:**
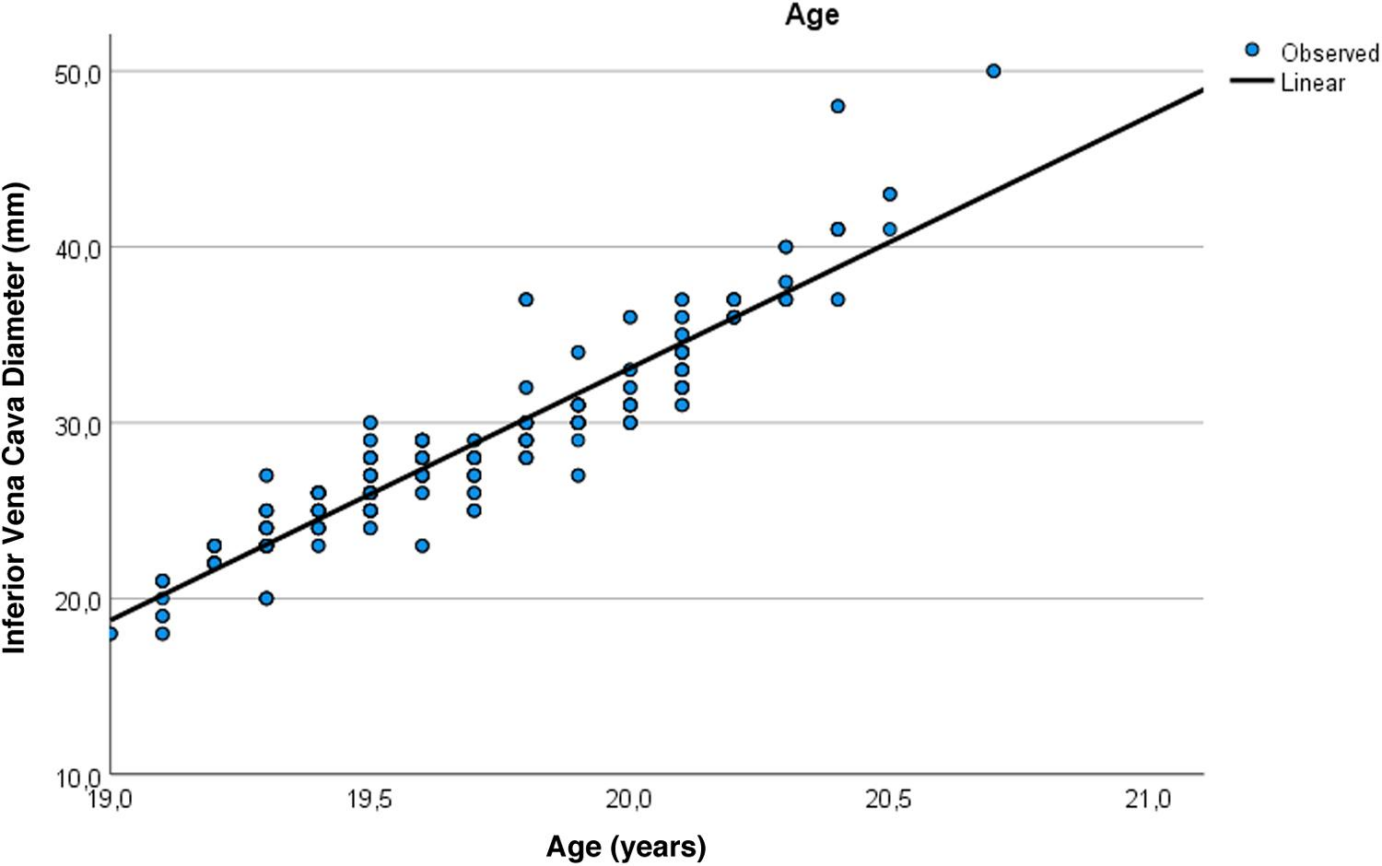
Visual summary of the very strong positive correlation between age and VCI expiratory diameter (*r* = 0.753; *p* < 0.001).

**Figure 2:**
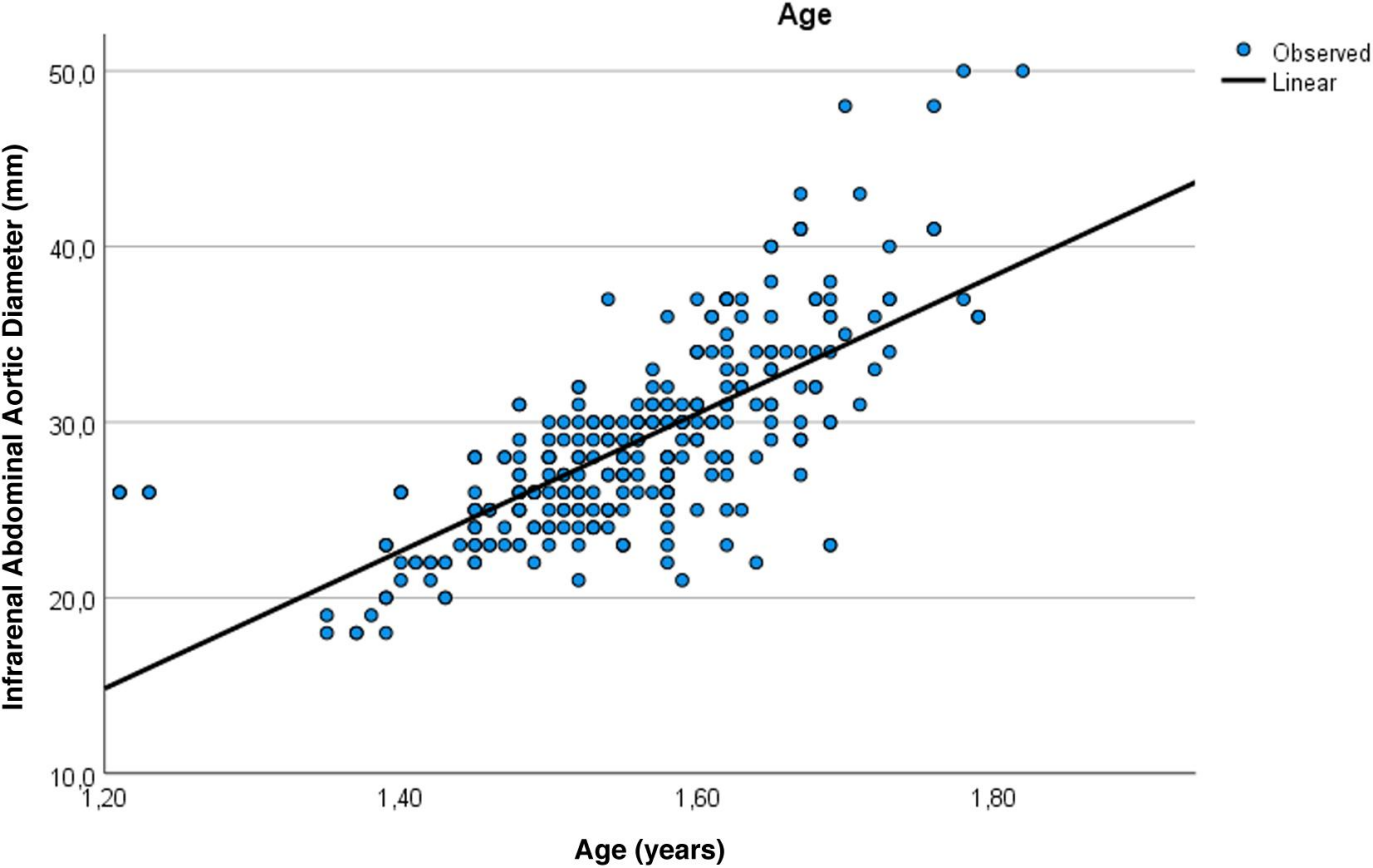
Visual summary of the very strong positive correlation between age and infrarenal abdominal aorta diameter (*r* = 0.935; *p* < 0.001).

**Figure 3:**
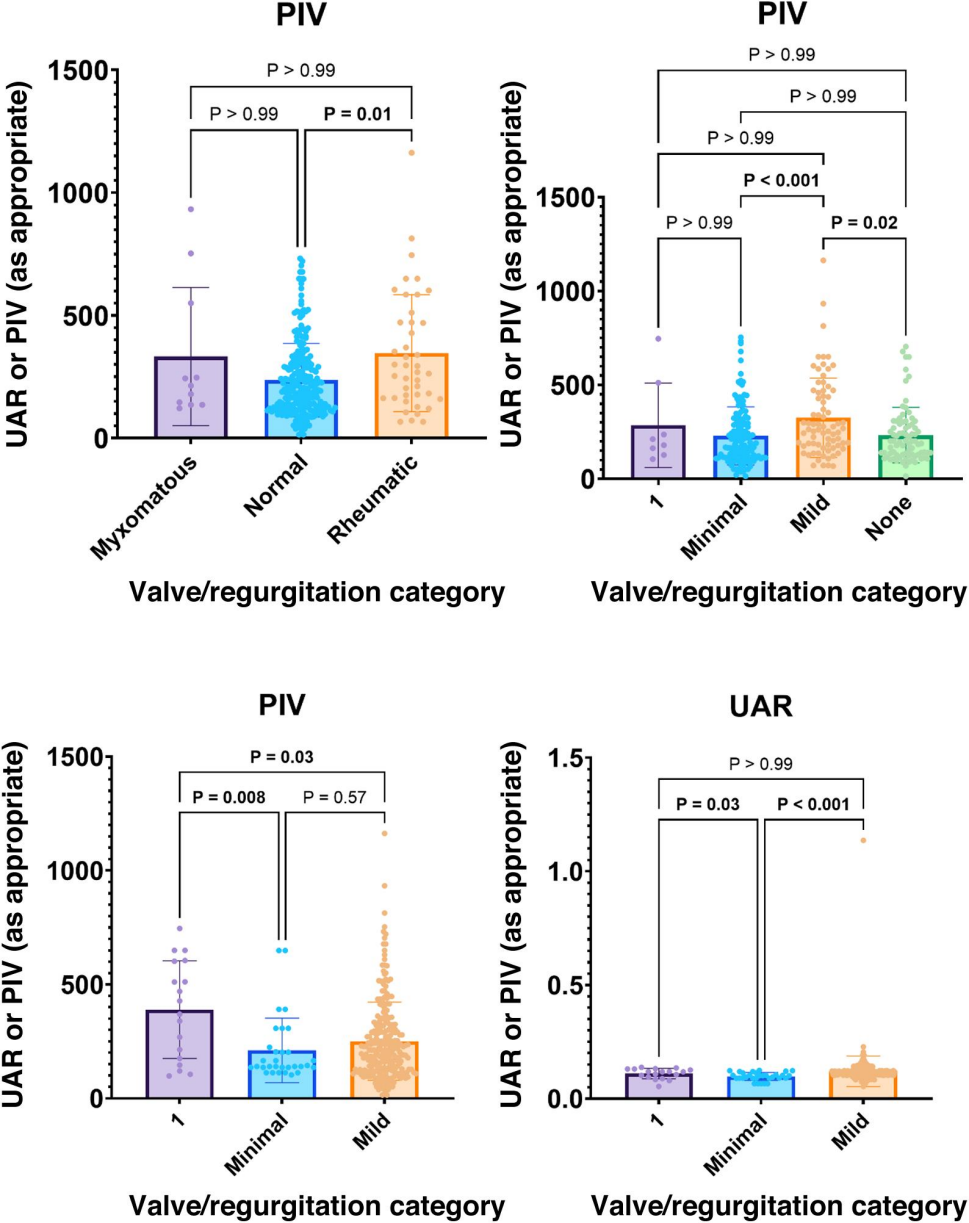
General summary of *post hoc* analyses and pairwise comparisons of UAR and PIV parameters that constitute significant differences according to valve pathology between groups. PIV, pan-immune inflammatory value; UAR, uric acid/albumin ratio.

Figure 1 graphically illustrates the strong positive correlation between age and expiratory IVC diameter (OUD + Suboxone vs. controls, *r* = 0.753; *p* < 0.001). Figure 2 demonstrates a very strong positive correlation between age and infrarenal abdominal aortic diameter (OUD + Suboxone vs. controls, *r* = 0.935; *p* < 0.001). Figure 3 presents the *post hoc* and pairwise comparisons of UAR and PIV values based on valvular pathology, highlighting significant intergroup differences.

## 4 Discussion

Opioid use disorder (OUD) remains a significant public health issue, directly affecting several million individuals in the United States (18–20). OUD impacts the lives of millions, both physically and emotionally. Buprenorphine/naloxone is an important pharmacological agent in the treatment of opioid use disorder (21). The combination of buprenorphine and naloxone (buprenorphine-naloxone) has been approved by the Food and Drug Administration (FDA) for detoxification and maintenance therapy in patients with opioid use disorder (22). The Drug Addiction Treatment Act has legalized the prescription of Schedule III drugs such as buprenorphine-naloxone by clinicians for the treatment of OUD (23, 24). Buprenorphine-naloxone was the first medication approved for outpatient treatment of opioid use disorder under the Drug Addiction Treatment Act of 2000 (22, 25). The sublingual tablet formulation was approved by the FDA in 2002 specifically for the treatment of opioid use disorder (22).

OUD is characterized by a strong motivation to obtain and use opioids despite significant adverse effects on health, occupational functioning, and interpersonal relationships (23). Buprenorphine-naloxone is a partial agonist used to reduce the frequency of full µ-opioid receptor agonist (e.g., heroin, fentanyl) use (24, 26). It attenuates the intensity of opioid-induced euphoria and reduces the incidence of associated adverse effects. Compared to placebo, buprenorphine-naloxone has been associated with improved retention in drug treatment programs, lower relapse rates, and reduced opioid-related overdose (27). Our study offers valuable insights into the cardiovascular effects of inflammatory biomarkers previously used in various studies—namely, the platelet-to-lymphocyte ratio (PLR) and the uric acid to albumin ratio (UAR)—with a specific focus on individuals undergoing buprenorphine/naloxone maintenance therapy, particularly in terms of left ventricular diastolic function and tissue Doppler findings.

Several studies have reported elevated uric acid levels in various cardiovascular diseases (28, 29). Uric acid, a product of purine metabolism, is recognized as a marker of oxidative stress and inflammation. Elevated serum uric acid levels have been linked to adverse cardiovascular outcomes, including heart failure and myocardial infarction. Our study suggests that when adjusted for albumin levels—which reflect the body's nutritional and inflammatory status—UAR may provide a more detailed assessment of the relationship between cardiac pathology and intravenous (IV) drug abuse. Albumin level is an established prognostic marker in a wide range of conditions, including cardiovascular disorders. Hypoalbuminemia is often observed in patients with severe inflammation and is associated with poorer clinical outcomes. By combining uric acid and albumin levels into a single ratio, UAR captures both oxidative stress and nutritional/inflammatory status. A study involving patients undergoing transcatheter aortic valve implantation (TAVI) demonstrated that UAR may predict early mortality (30). Another study on acute pericarditis patients emphasized the association of UAR with clinical severity, recurrence risk, and its potential role in improving outcomes (31). In our study, UAR levels were significantly higher among individuals with opioid use disorder (OUD) who are maintained on Suboxone therapy. Furthermore, an increasing degree of pulmonary regurgitation was associated with significantly higher UAR values. Elevated UAR in IV drug users suggests the presence of active inflammatory processes potentially linked to underlying cardiac pathology in this population.

Inflammation and thrombosis are closely interconnected and mutually dependent processes. Neutrophils, in particular, play a critical role in both atherosclerosis and thrombus formation. While neutrophil counts reflect the duration of inflammation, lymphocyte levels represent immune-regulatory mechanisms (32). Inflammatory processes increase neutrophil, monocyte, and platelet counts, while simultaneously decreasing lymphocyte levels (33). A post-acute inflammation drop in lymphocytes has been associated with adverse cardiovascular outcomes. Additionally, platelets contribute to both acute and chronic inflammatory processes involved in coronary artery disease (CAD) (34).

Wu et al. demonstrated that high PIV is associated with worse outcomes in various cardiovascular conditions and reflects an underlying heightened inflammatory state (35). The pan-immune-inflammation value (PIV), which incorporates four key hematological parameters—neutrophils, monocytes, platelets, and lymphocytes—has recently been developed as a marker to assess the severity of inflammation (33, 36–44). Recent studies have confirmed the prognostic significance of PIV in several inflammatory conditions (45–47). A study on patients with ST-segment elevation myocardial infarction found that PIV had superior predictive value for mortality compared to the neutrophil-to-lymphocyte ratio (NLR) and the platelet-to-lymphocyte ratio (PLR) (48). Moreover, Wu et al. also demonstrated that PIV is a reliable indicator for predicting cardiovascular mortality (49). According to Çetinkaya et al., despite a cut-off value of 568.2, PIV could predict severe coronary lesions with 91% sensitivity and 81.1% specificity (32). The findings of Özcan Çetin et al. (2017) and Kalyoncuoğlu et al. (2020), showing that elevated WBC and PIV levels are indicative of severe inflammatory processes, are consistent with our results (36, 50).

In our study, PIV levels were significantly higher among individuals with opioid use disorder (OUD) who are maintained on Suboxone therapy. Evaluation of affected valvular structures and the severity of regurgitation showed a significant association between elevated PIV values and the presence of myxomatous or rheumatic mitral valve pathology compared to structurally normal valves. A significant relationship was also observed between the presence of mild mitral regurgitation and the highest PIV values. Patients with mild mitral regurgitation had higher PIV levels than those with trace or no regurgitation. Moreover, as the severity of pulmonary regurgitation increased, PIV levels also increased significantly. Elevated PIV levels among individuals with opioid use disorder (OUD) who are maintained on Suboxone therapy suggest the involvement of inflammatory processes that may contribute to cardiac pathologies in this population.

Both e′ and E/e′ retained their predictive value even after adjusting for traditional cardiovascular (CV) risk factors as represented by the ACC/AHA ASCVD risk score. For each one-unit decrease in e′, there was a 22% increase in the risk of cardiovascular death, while each one-unit increase in E/e′ was associated with a 12% increased risk in adjusted analyses. Tissue Doppler-derived indices have become an integral component of echocardiographic evaluation of left ventricular (LV) diastolic function and are endorsed by current clinical guidelines. These include the measurement of peak e′ velocity at both the septal and lateral mitral annuli and the calculation of their average. This methodology was adopted in our study and underscores the importance of incorporating these measurements into standard echocardiographic evaluations. Peak e′ velocity is relatively preload-independent and is a highly reproducible parameter (51). Compared to LV ejection fraction and LV mass, tissue Doppler e′ and E/e′ are easier to obtain and more reproducible (52). It is well-established that LV relaxation and e′ deteriorate with age, potentially due to progressive myocardial fibrosis (53). Human studies have shown a strong correlation between e′ and interstitial fibrosis (54). The robust association between e′ and cardiovascular mortality may be due to its superior sensitivity compared to LV mass or systolic tissue velocity (s′). Both e′ and s′ decline with age, but the inverse correlation between age and e′ is much steeper, supporting this hypothesis. Pathologies such as hypertension, diabetes, and coronary artery disease—which are known contributors to early coronary and cerebrovascular events—are also linked to myocardial fibrosis, potentially increasing the risk of heart failure or arrhythmic cardiac death. Vascular wall alterations occur in parallel with cardiac changes, and a lower e′ velocity may serve as an indicator of more severe fibrosis in the arterial walls, which in turn contributes to increased vascular stiffness and a higher risk of plaque rupture (55–58).

Studies have demonstrated a strong association between arterial stiffness and diastolic dysfunction, where increased arterial stiffness is linked to impaired left ventricular (LV) relaxation and elevated E/e′ ratios (59). This relationship is particularly important in conditions such as heart failure with preserved ejection fraction (HFpEF) and atrial fibrillation (AF), where combined reductions in both e′ and s′ velocities are predictive of up to a 12-fold increase in adverse clinical outcomes, including heart failure decompensation, ischemic stroke, and cardiovascular mortality (60).

Moreover, lifestyle factors such as obesity, hypertension, and physical inactivity contribute to systemic inflammation, which promotes fibroblast activation and collagen deposition, thereby exacerbating myocardial fibrosis and diastolic dysfunction. Addressing these modifiable lifestyle factors is crucial for the prevention and management of diastolic dysfunction (61).

In our study, there were no significant differences in mitral E and A velocities, s′, septal and lateral e′ velocities, or lateral E/e′ ratios between individuals with individuals with opioid use disorder (OUD) who are maintained on Suboxone therapy and the control group. However, septal A′ and lateral A′ velocities, as well as the septal E/e′ ratio, were significantly higher in the individuals with opioid use disorder (OUD) who are maintained on Suboxone therapy group compared to controls. The elevated septal E/e′ ratio observed in individuals with opioid use disorder (OUD) who are maintained on Suboxone therapy is particularly noteworthy, as it may indicate the presence of diastolic dysfunction in this population.

The significance of the septal E/e′ ratio in both univariate and multivariate logistic regression analyses underscores its potential clinical relevance.

Additionally, the significantly larger diameter of the inferior vena cava (IVC) observed in the individuals with opioid use disorder (OUD) who are maintained on Suboxone therapy group, along with its significance in univariate logistic regression analysis, is of interest. A strong positive correlation was also noted between IVC diameter and infrarenal abdominal aortic diameter with age. Our data do not allow us to distinguish whether the observed cardiovascular findings arise primarily from chronic opioid use disorder (OUD) itself or from factors associated with long-term treatment exposure. As a retrospective study, our design does not permit causal interpretation.

## 5 Limitations

This study has several limitations that should be taken into consideration. First, its retrospective design may introduce selection and data collection biases, potentially affecting the generalizability of the findings. Second, the relatively small sample size and the single-center nature of the study may not reflect variations in clinical practice or patient characteristics across different settings. The duration of OUD before initiation of maintenance therapy was not available, which limits causal interpretation.

Finally, although the Pan-Immune-Inflammation Value (PIV) and Uric Acid-to-Albumin Ratio (UAR) show promise in detecting cardiac pathologies, the lack of prospective validation and integration with other clinical variables highlights the need for further research to confirm these findings and to assess their practical utility in routine clinical practice.

## 6 Conclusion

Our study demonstrates that individuals with opioid use disorder (OUD) who are maintained on prescribed Suboxone therapy exhibit distinct patterns of valvular abnormalities, right-sided cardiac enlargement, and impaired diastolic function. The concurrent elevation of PIV and UAR suggests that systemic inflammation and oxidative stress may contribute to the development of these structural cardiac alterations. These findings highlight the potential utility of PIV and UAR as accessible, low-cost biomarkers for early cardiovascular risk assessment in this population. While our results emphasize the importance of routine echocardiographic evaluation in individuals with long-standing OUD, larger prospective studies are required to clarify causality and to further validate the prognostic significance of these biomarkers.

## Data Availability

The raw data supporting the conclusions of this article will be made available by the authors, without undue reservation.
